# The TIPS family psychoeducational group work approach in first episode psychosis and related disorders: 25 years of experiences

**DOI:** 10.1111/eip.13591

**Published:** 2024-07-16

**Authors:** Johannes H. Langeveld, Kristin Hatløy, Wenche ten Velden Hegelstad, Jan Olav Johannessen, Inge Joa

**Affiliations:** ^1^ TIPS Centre for Clinical Research in Psychosis Stavanger University Hospital Stavanger Norway; ^2^ Faculty of Health University of Stavanger Stavanger Norway; ^3^ Faculty of Social Sciences University of Stavanger Stavanger Norway

**Keywords:** early intervention, family, psychosis

## Abstract

**Aim:**

The aim of this paper is to present 25 years of clinical experience with family psychoeducation (FPE) work at Stavanger University Hospital in Norway, highlighting the lessons learned in overcoming implementation barriers in publicly funded specialized mental health care.

**Methods:**

This retrospective analysis reviews the integration and sustainability of FPE work within the hospital's standard treatment protocols for psychosis, tracing its origins from the Early Treatment and Intervention in Psychosis (TIPS) study (1997–2000) to its current application. The paper examines key strategies for successful implementation, including staff training and resource allocation, as emphasized by international research.

**Results:**

Stavanger University Hospital has successfully implemented and maintained both multi‐ and single‐family FPE approaches over the past 25 years. Initially part of the TIPS study, FPE has been integrated into routine clinical practice for treating psychosis and has recently been extended to families of patients with other severe mental disorders. The sustained success at Stavanger University Hospital is attributed to consistent staff training and the prioritization of sufficient resource allocation.

**Discussion:**

The successful and sustainable integration of FPE at Stavanger University Hospital is relatively unique. International guidelines recommend FPE for psychosis, but its implementation remains inconsistent globally, despite over 50 years of supporting evidence. The hospital's experience underscores the critical role of continuous training and dedicated resources in embedding FPE into regular clinical practice. These findings suggest that addressing these areas can significantly enhance the uptake of FPE in other clinical settings.

**Conclusion:**

The 25‐year experience at Stavanger University Hospital demonstrates that with appropriate training and resources, FPE can be successfully integrated and sustained within standard mental health care practices. This case study provides valuable insights for other institutions aiming to implement FPE and improve treatment outcomes for patients with severe mental disorders.

## 1 INTRODUCTION

The development of psychosis usually begins in adolescence or young adulthood (NHS England, 2016) and often disrupts important developmental tasks in education, the pursuit of a career, finding a partner, and establishing a social life (World Health Organisation, 2022). Psychosis further affects the whole family of the young person, as family members often constitute the person's main support system (Addington et al., 2005).

Early support for families and significant others is associated with better prognosis (McFarlane, 2016). Psychoeducational approaches in family work, either single families or multiple families in a group, with or without the person suffering from psychosis, is recommended in most international guidelines for the treatment of psychosis (American Psychiatric Association, 2020; Helsedirektoratet, 2013; Kuipers et al., 2014). Nevertheless, it is not commonly implemented in clinical practice (Hestmark et al., 2020; Hestmark et al., 2021; Hestmark et al., 2023; Selick et al., 2017).

## 2 THE DEVELOPMENT OF FAMILY WORK FROM AN INTERNATIONAL PERSPECTIVE

Since the early 1950s, there have been various attempts to study interactions in families where one member suffers from severe mental illness. In 1953, Abrahams and Veron (1953) described a group work project in which several girls suffering from schizophrenia met together with their mothers. Family work from the early 60s became defined as a partnership between the patient, family members, and health professionals (Alanen et al., 2009). During the 60s, the expressed emotion (EE) concept was introduced as a way to understand the emotional environment created by the interactions between individuals, particularly within families (Brown & Rutter, 1966). Moving into the 1970s, the notion arose that high levels of EE within a family environment, comprising criticism, hostility, and emotional overinvolvement, can be linked to various mental health issues, including psychosis (Brown et al., 1972). Consequently, family interaction patterns can affect the duration of hospitalization and frequency of readmissions of psychiatric patients. However, few inpatient units offered treatment programmes that involved families. Therefore, a treatment concept was developed that combined the acceptance of a primary focus on the patient's illness with the recognition of the importance of family factors (Anderson, 1977). In the 1980s the partnership concept from the 60s and 70s was further developed (Anderson, 1986), and single‐family interventions for patients with psychosis were introduced in England (Dixon et al., 2001; Falloon, 2003; Falloon et al., 1984; Goldstein & Doane, 1982; Held & Falloon, 1999; Leff, 1979; Miklowitz, 2012).

In the next decennium, McFarlane et al. (2003) refined the interventions into a structured programme including both single and multiple families. This was in part based on the work by Anderson (1986). Another 10 years later, variants of this programme were developed for bipolar disorders and ultra‐high risk for psychosis states (Leung et al., 2021; Miklowitz, 2012; Miklowitz et al., 2018; Miklowitz et al., 2021; Minuchin et al., 2014; Worthington et al., 2021). Common to these modified programmes were (1) a focus on mental health literacy and problem‐solving strategies (2) the continuous availability of family support and (3) a treatment duration between 6 months and 5 years (Dixon et al., 2001; Dixon et al., 2010; McFarlane et al., 2003). In our time, online social networking programmes for caregivers of young people with psychosis have been designed. Such programmes integrate expert and peer moderation with evidence‐based psychoeducation in a web‐based solution. For instance, the Relatives Education and Coping Toolkit (REACT) is a guided self‐management intervention for relatives of persons with recent onset psychosis (Lobban et al., 2011; Romm et al., 2020).

A more recent review article (McFarlane, 2016) presents updated research on FPE work. The included studies indicated that interventions including families and containing psychoeducation were effective in reducing the number of symptom relapses and hospitalizations. Patients gained increased social skills, improved their drug treatment adherence, and families experienced an increase in their quality of life (Morin & Franck, 2017; Pharoah et al., 2010). Psychoeducation is particularly effective in raising relatives' mental health literacy and coping skills (Sin & Norman, 2013).

Another review and meta‐analysis (Claxton et al., 2017) also indicated that family interventions are effective for early psychosis service users and their relatives, reducing relapse and carer distress. A prospective, quasi‐experimental but relatively small cohort study concluded that single‐family interventions might be equally effective as multi‐family interventions in a FEP programme (Haahr et al., 2020). The most salient advantage of multi‐family compared to single‐family interventions is the socialization process in these groups, which may result in shared experiences and the perception of not being alone in struggling with learning how to live with and support the family member who has mental health (McFarlane, 2004). On the other hand, single‐family interventions have the advantage of meeting the needs of the families more individually (Haahr et al., 2020).

FPE aims at improving functional outcomes and quality of life as well as at reducing family stress and strain (Anderson, 1986; Iyer et al., 2011; McFarlane et al., 2003; Nilsen et al., 2014; Thorsen et al., 2006). Based on the notion that reducing EE and promoting supportive family environments can be beneficial in improving outcomes for individuals with psychosis (Wearden & Tarrier, 2000), the overall focus is on lowering EE, particularly negative emotions and criticism, to help the family gain a hopeful perspective on the future, counting in the vulnerability for psychosis. Further, the focus lies on solving everyday challenges in a structured and constructive way (Breitborde et al., 2011), and thus to reduce stress. Reducing stigma is also important. Unfortunately, there is still stigma attached to severe mental illness, which often results in self‐stigma on part of both families and patients.

In FPE groups, patient participation is strongly preferred. FPE group work provides education about causes, course, vulnerability, and treatment of psychosis. Every group is guided by two group leaders who have undergone training in the method. The method is manualized and comprises bi‐weekly meetings. The families practice problem‐solving and healthy communication skills in the group. To allow families to adjust their communication and to gain better problem‐solving strategies, the groups should last for a minimum period of 9–12 months (Dixon et al., 2001; Dixon et al., 2010; McFarlane et al., 2003; Murray‐Swank & Dixon, 2004).

**FIGURE 1:**
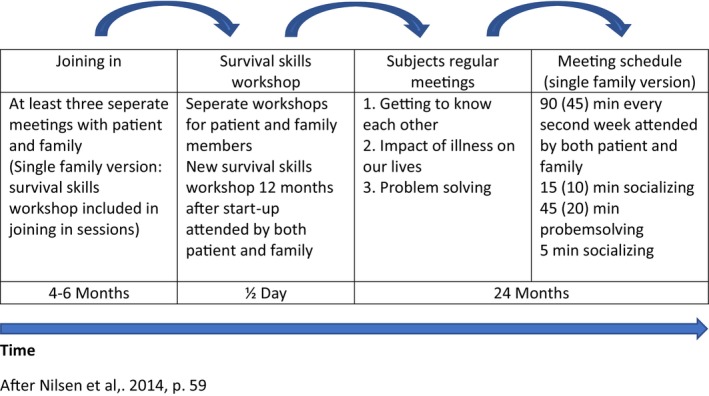
The structure of psychoeducational family interventions.

As is depicted in Figure 1, the FPE method (McFarlane et al., 1995) comprises three stages: “The joining in” period, survival skills workshop and the regular meetings as shown in Figure 1. A more detailed description of the method is described elsewhere (Rossberg et al., 2010).

### 2.1 The FPE group work at TIPS in Norway

The Stavanger University Hospital branch of the early Treatment and Intervention in Psychosis (TIPS) study in Norway and Denmark (Friis et al., 2005; Johannessen et al., 2001) has a 25‐year history and might serve as an example on how implementation barriers have been overcome and how FPE has been integrated in a long‐term clinical setting. When TIPS started in 1996, the main goal was to achieve early detection of and intervention in psychosis. To implement evidence‐based psychoeducational family work in ordinary clinical services for FEP, was an important secondary goal. In 1996, the first multi‐family groups were set up. Eligible families were those having a family member with FEP meeting criteria for the TIPS study (Johannessen et al., 2001; Rossberg et al., 2010). The FPE group work leaders were trained in the method following the translated manual by McFarlane et al. (1995). The duration of the FPE intervention was set to 2 years of bi‐weekly meetings outside office hours to facilitate participation for working family members. As time passed, the intervention gained momentum and the number of group leaders as well as participants grew. Having hospital management supporting the intervention remained important to secure the needed resources to maintain and develop the programme. As a parallel process, resources were made available to set up an “FPE school” where new group leaders were trained in the method and had regular supervision. This group leader training has since 1997 been a permanent educational offer in the services. TIPS FPE is now cooperating closely with TIPS South‐East (Oslo) and is part of the Norwegian National Network for Family Cooperation.

### 2.2 Adaptations of the FPE approach to specific groups

The first implementation step was to establish a FPE group work for psychosis. Subsequently, we aimed to offer this approach to other patient groups as well, and as the development in the field of psychosis research and treatment shifted towards ultra‐high risk for psychosis, the psychoeducational family work in Stavanger followed. Necessary adaptations have been made to meet the specific needs of this group (Sviland et al., 2017). For instance, ultra‐high risk for psychosis has a different course and prognosis compared with FEP, as only about 20% of those suffering from it will have developed a first episode of psychosis within 2 years after start of treatment (Joa et al., 2021). Targeting the symptoms and challenges at hand, and not only the risk of psychosis per se, is important considering the younger age of most of these patients (O'Brien et al., 2007). Indeed, groups for adolescents and couples (mixed psychotic and affective disorders) are now running. For all groups, only minor adaptations of the programme were necessary.

In Table 1, we present a number of groups and participants for the different patient categories from 1997 to 2022 in the Stavanger area (target population of ~400 000 inhabitants).

**TABLE 1:** Number of groups and participants for different patient categories (1997–2022) in the Stavanger area (population: 400 000).

Multi‐family groups 1997–2022	# Groups	# Patients	# Relatives	Group leaders
Multi‐family groups psychosis/affective youth (13–18 years)	25	102	176	
Multi‐family groups psychosis families (19 years–>)	53	225	384	
Multi‐family groups affective families (19 years–>)	16	64	108	
Multi‐family groups psychosis couples	10	40	40	
Multi‐family groups affective couples	26	114	114	
Relatives groups psychosis/drugs	10	43	83	
Sum	140	587	905	444

*Note*: This table presents the number of groups, patients, relatives, and group leaders for different patient categories in the Stavanger area from 1997 to 2022.

Table 2 shows the present activities of the FPE work in the Stavanger area.

**TABLE 2:** Active groups as per August 2023.

	Groups	Patients	Relatives
Multi‐family groups psychosis/affective, youth (13–18 years)	3	12	23
Multi‐family groups, psychosis/affective families (19 years–>)	6	24	49
Multi‐family groups, psychosis/affective couples	5	21	21
Relatives groups, psychosis/drugs	1	6	9
Partners groups, dementia	4	32	32
Single‐family groups psychosis/affective	10	9	17
REACT‐NOR guidance for relatives psychosis/affective	38	35	35

*Note*: The number of groups, patients, relatives, and group leaders for different patient categories in the Stavanger area as per august 2023.

Data about recruitment of relatives are not included in the tables. Overall, for each two patients, three relatives participate (1.5 relative per patient).

## 3 IMPLEMENTATION BARRIERS AND FACILITATORS IN PSYCHOEDUCATIONAL FAMILY WORK

Despite the evidence for the benefits of family interventions, and their frequent application in early intervention programmes around the world, these programmes are not widely implemented in ordinary clinical settings (Eassom et al., 2014). The travel from project to ordinary clinical service has been challenging.

An Australian study (Harvey & O'Hanlon, 2013a; Harvey & O'Hanlon, 2013b) on the implementation of FPE interventions in people with schizophrenia and other psychotic disorders highlights the importance of intensive implementation support. This includes dedicated and protected time for FPE workers and active management facilitation of organizational change to support the new practice.

A review study on implementation barriers and facilitators identified 4402 unique studies that included terms related to early onset, implementation, and family work (Selick et al., 2017). The seven studies meeting the inclusion criteria for the final review particularly highlighted the importance of staff access to training and resources for providing family support.

A recent study (Hansson, Romøren, Pedersen, et al., 2022) describes a Norwegian cluster randomized implementation study. It included 16 months of follow‐up and a qualitative investigation of organization‐level barriers to implementation. Three themes were identified: (1) Lack of shared knowledge, perceptions, and practice (2) Lack of routines (3) Lack of resources and coordination. Conversely, results suggested four organizational level facilitators: (1) Whole‐ward approach (2) Appointed and dedicated roles (3) Standardization and routines (4) External implementation support. At the clinical level, the study identified two additional facilitating factors: (5) Understanding, skills, and self‐efficacy and (6) Awareness and positive attitudes among mental health professionals. The investigators conclude that providing training in FPE to all clinical staff, followed by clinicians gaining experience with family involvement, may lower or dissolve core barriers.

### 3.1 Overcoming implementation barriers in Stavanger

In Stavanger, Norway, participants are mostly referred to FPE through the TIPS early detection system, as part of the hospital's “psychosis treatment line” (Johannessen et al., 2001). Between 1997 and 2005, the TIPS studies only included FEP patients. As eligibility criteria were expanded to other disorders, routines for referring other participants were developed and implemented for each diagnosis category. After assessment using the Structured Clinical Interview for DSM‐IV (SCID‐I) (First et al., 1997) or the Structured Interview for DSM‐5 (First et al., 2016), diagnoses are determined based on DSM criteria. During the last 4 years (2020 through 2023), we received referrals for FPE group work to 649 patients and their families. Of these, 122 (19%) were not eligible (wrong diagnosis, not having any family, previously having received the intervention, having moved out of the catchment area) and 176 (27%) declined to participate, leaving 351 (54%) patients and their families to receive either multi‐ or single‐family work, the REACT web‐based intervention, or any combination of these.

The next section presents a discussion of the TIPS approach to the implementation of FPE, following the identified facilitators by Hansson, Romøren, Weimand, et al. (2022).

1. A *whole‐ward approach*: The approach at TIPS followed this strategy to a wide extent. Not only one single ward, but the entire clinic was included in the implementation process. FPE was provided across all units, wards and outpatient clinics for patients with psychosis. However, groups were run outside office hours, implying extra work for staff. FPE group workers were therefore offered financial compensation for overtime and an annual one‐week extra holiday leave.
2. Hansson, Romøren, Weimand, et al. (2022) also pointed to the importance of having *clear and dedicated roles* in a local implementation team, and a family work coordinator. TIPS established a specialized family outpatient clinic employing two full‐time FPE coordinators. This outpatient family clinic is serving all levels and hospital units in our catchment area by responding to referrals, planning start‐ups of new FPE groups, follow‐up of ongoing groups, group leader recruitment, training, and supervision.
3. *Standardization and routines*: The Stavanger TIPS family outpatient clinic approach includes provision of written procedures and information leaflets, documentation templates, and systems for routinely developing crisis plans and inviting all family members and other relevant persons in the network to evening seminars and courses. The outpatient clinic schedules weekly meetings with the TIPS team and the inpatient wards, resulting in optimal communication at all levels.
4. *External implementation support*: The two full‐time FPE group work coordinators initiate and organize the multi‐ and single‐family groups and are responsible for quality assurance and the organizing of training and supervision for FPE group leaders and use of the web‐based REACT support programme.
5. *Understanding, skills, and self‐efficacy among mental health professionals*: Group leaders are recruited from the mental health services. Their participation is based on their qualification as at least at bachelor level educated mental health care workers and on personal interest. They receive extensive training during working hours, facilitated by hospital management.
6. *Awareness and positive attitudes among mental health professionals*: The TIPS system provides information and easy access to all hospital staff in mental health care in the catchment area. Smooth and flexible communication, swift reply to all referrals and questions, and accessibility, have been proven key to the positive attitudes towards and awareness of the intervention.

Additionally, in line with other recommendations (Harvey & O'Hanlon, 2013a; Harvey & O'Hanlon, 2013b), the time that group workers spend on FPE work is dedicated and protected from other tasks. This requires facilitation and organization at management levels, which TIPS has succeeded in obtaining.

## 4 DISCUSSION

Although some studies question the consistency and generalizability of EE research and present alternative models to explain the influence of family interactions on the development and course of psychosis (e.g., Read & Gumley, 2008), our experience highlights the practical utility and relevance of EE in a psychoeducational family group work setting.

Tailoring family psychoeducation (FPE) groups according to the patient's age and family structure is a strength of the TIPS approach. By recognizing the diversity of family dynamics and relationships, TIPS can effectively customize the teaching and support provided to meet the unique needs of patients and their family members.

The use of both multi‐family and single‐family group formats in our FPE programme reflects a flexible and inclusive approach to addressing the diverse needs of patients and their families. While participants may initially be hesitant to share their experiences with other families, our experience suggests that both formats can be equally effective in fostering a sense of support and connection among participants as the group work progresses.

The time horizons for FPE group work varies (Dixon et al., 2001; Harvey & O'Hanlon, 2013a; Harvey & O'Hanlon, 2013b). Our single‐family and REACT interventions have a duration of respectively 9–12 and 4–6 months. We choose to extend the duration of our multi‐family FPE groups to 2 years, which offers the advantage of time. Building trust and rapport within a group takes time, especially in environments like FPE groups where participants may be sharing personal experiences and vulnerabilities.

Organizing FPE work as an independent outpatient clinic with dedicated staff as we do, promote continuity of care, flexibility, and tailored support for patients and their families across the spectrum of the disorder's phases. In the midst of chaos, FPE programmes provide a sense of predictability and stability. Indeed, during the COVID epidemic, families who were in contact with FPE experienced health care and support to be more available compared to families who were not (Aminoff et al., 2022).

Having FPE group workers with diverse positions within the hospital setting facilitates the integration of FPE with clinical practice by providing multidisciplinary expertise, ensuring clinical relevance, promoting seamless referral and collaboration, enabling the practical application of skills, supporting a holistic approach to care, and fostering a continuous quality improvement process. This collaborative approach enhances the effectiveness and impact of FPE in supporting families affected by mental health disorders. The challenge of frequent reorganizations and turnover of therapists can indeed pose obstacles to maintaining awareness and continuity of services. To address this challenge and ensure that all relevant units stay informed about the work, we have developed comprehensive documentation and training materials that provide an overview of the TIPS Outpatient Family Clinic and FPE programme. New practitioners can go through these materials to familiarize themselves with the services, including their purpose, structure, and approach. Further, the FPE group leaders serve as resources for questions and information about the TIPS Outpatient Family Clinic and FPE at their respective mental health care units. This helps facilitate communication and collaboration between the clinic and other areas of the hospital.

We are still improving the systematic recruitment of patients and family members for the FPE, as this is essential for its success and effectiveness. By leveraging the involvement of more group leaders who have gained management positions, FPE can further enhance recruitment efforts. This approach ensures that patients and family members receive timely access to the necessary support and resources.

We have extended our FPE work to benefit families affected by a range of mental health diagnoses beyond psychoses. In our experience, this expansion has the potential to improve outcomes, enhance family support, and promote recovery across diverse clinical populations. This experience aligns with findings from a systematic literature review suggesting similarities between FPE in psychosis and bipolar disorder (Mansfield et al., 2012), particularly regarding the challenges related to reduced reality understanding and insight that patients with manic and psychotic symptoms often share. However, studies on FPE for other diagnoses remain scarce.

Balancing the capacity of group work in our FPE programme can be challenging, especially when considering the need to accommodate new patients and their relatives while ensuring that group leaders remain engaged and committed in their roles. In our experience, ongoing recruitment and training strategies and mentorship are key components of a successful approach to managing capacity challenges in FPE group work.

Finally, managing the time window for starting up new groups in a FPE programme can be a complex challenge, especially when balancing the need to minimize waiting times for candidate families with the desire to maintain an optimal group size which is between six and eight families (Dixon et al., 2001).

## 5 CONCLUSION

FPE group work has become an exciting new avenue of psychosis treatment. Over 25 years, the FPE group work at TIPS Hospital has prospered, matured, and grown. We have broadened the scope of the FPE work towards other diagnoses than psychosis and towards including modern, digital tools. We indicated some success factors for implementation and some challenges we still meet. Both patients, their relatives, and the group workers experience the group work as meaningful. If these findings are robust to future analyses with a more stringent design, it would strengthen the evidence of the method, which may in turn increase its implementation.

## FUNDING INFORMATION

This research received no specific grant from any funding agency in the public, commercial, or not‐for‐profit sectors.

## CONFLICT OF INTEREST STATEMENT

The authors certify that they have no affiliations with or involvement in any organization or entity with any financial interest (such as honoraria; educational grants; participation in speakers' bureaus; membership, employment, consultancies, stock ownership, or other equity interest; and expert testimony or patent‐licensing arrangements), or non‐financial interest (such as personal or professional relationships, affiliations, knowledge or beliefs) in the subject matter or materials discussed in this manuscript.

## Data Availability

The data that support the findings of this study are available on request from the corresponding author. The data are not publicly available due to privacy or ethical restrictions.
